# Supplementary material for: Factors associated with overweight and obesity among women of reproductive age in Cambodia: Analysis of Cambodia Demographic and Health Survey 2021–22
Source: PLOS Glob Public Health. 2024 Jan 31;4(1):e0002537. doi: 10.1371/journal.pgph.0002537 (PMC10830042; doi:10.1371/journal.pgph.0002537)
Supplement: S1 Table — (DOCX) [file pgph.0002537.s001.docx]

**S1 Table**. Results of checking multicollinearity using Variance Inflation Factor (VIF)

| **Variables** | **VIF** |
| --- | --- |
| Woman’s age | 2.21 |
| Number of children born | 2.20 |
| Marital status | 1.60 |
| Household wealth index | 1.59 |
| Education | 1.48 |
| Place of residence | 1.30 |
| Contraceptive use | 1.16 |
| Geographical regions | 1.09 |
| Occupation | 1.07 |
| Smoking | 1.06 |
| Alcohol consumption | 1.04 |
| Mean VIF | 1.44 |
