# Supplementary material for: Factors associated with overweight and obesity among women of reproductive age in Cambodia: Analysis of Cambodia Demographic and Health Survey 2021–22
Source: PLOS Glob Public Health. 2024 Jan 31;4(1):e0002537. doi: 10.1371/journal.pgph.0002537 (PMC10830042; doi:10.1371/journal.pgph.0002537)
Supplement: Attachment — Submitted filename: Response to Reviewers.docx [file pgph.0002537.s003.docx]

Authors’ point-by-point responses to the editor and reviewers

PGPH-D-23-01290

Factors Associated with Overweight and Obesity among Women of Reproductive Age in Cambodia: Analysis of Cambodia Demographic and Health Survey

PLOS Global Public Health

Dear Dr. Um,

Thank you for submitting your manuscript to PLOS Global Public Health. After careful consideration, we feel that it has merit but does not fully meet PLOS Global Public Health’s publication criteria as it currently stands. Therefore, we invite you to submit a revised version of the manuscript that addresses the points raised during the review process.

==============================

The review's comment about a Directed Acyclic Graph (DAG) seems like something that could be a valuable addition to your exposition, and I would like to see your revision include a DAG that represents the causal relationships that you hypothesize are present between the factors you have included in your analysis. I believe that this DAG will also provide a crisp framework for you to address the reviewer's question about what specifically your paper adds to the existing knowledge.

==============================

Please submit your revised manuscript by Nov 18 2023 11:59PM. If you will need more time than this to complete your revisions, please reply to this message or contact the journal office at globalpubhealth@plos.org. When you're ready to submit your revision, log on to https://www.editorialmanager.com/pgph/ and select the 'Submissions Needing Revision' folder to locate your manuscript file.

Please include the following items when submitting your revised manuscript:

A rebuttal letter that responds to each point raised by the editor and reviewer(s). You should upload this letter as a separate file labeled 'Response to Reviewers'.

A marked-up copy of your manuscript that highlights changes made to the original version. You should upload this as a separate file labeled 'Revised Manuscript with Track Changes'.

An unmarked version of your revised paper without tracked changes. You should upload this as a separate file labeled 'Manuscript'.

Guidelines for resubmitting your figure files are available below the reviewer comments at the end of this letter.

We look forward to receiving your revised manuscript.

Kind regards,

Abraham D. Flaxman, Ph.D.

Academic Editor

PLOS Global Public Health

**Journal Requirements:**

1. Please provide separate figure files in .tif or .eps format.

For more information about figure files please see our guidelines:

https://journals.plos.org/globalpublichealth/s/figures

https://journals.plos.org/globalpublichealth/s/figures#loc-file-requirements

Thanks. We have provided the separate figure files in.tif format which are labeled Fig 1, and Fig 2 on the revised submitted.

Additional Editor Comments (if provided):

[Note: HTML markup is below. Please do not edit.]

Reviewers' comments:

Reviewer's Responses to Questions

**Comments to the Author**

1. Does this manuscript meet PLOS Global Public Health’s publication criteria? Is the manuscript technically sound, and do the data support the conclusions? The manuscript must describe methodologically and ethically rigorous research with conclusions that are appropriately drawn based on the data presented.

Reviewer #1: Partly

We have revised it accordingly.

2. Has the statistical analysis been performed appropriately and rigorously?

Reviewer #1: No

Thank. We have revised it accordingly.

3. Have the authors made all data underlying the findings in their manuscript fully available (please refer to the Data Availability Statement at the start of the manuscript PDF file)?

The PLOS Data policy requires authors to make all data underlying the findings described in their manuscript fully available without restriction, with rare exception. The data should be provided as part of the manuscript or its supporting information, or deposited to a public repository. For example, in addition to summary statistics, the data points behind means, medians and variance measures should be available. If there are restrictions on publicly sharing data—e.g. participant privacy or use of data from a third party—those must be specified.

Reviewer #1: Yes

4. Is the manuscript presented in an intelligible fashion and written in standard English?

PLOS Global Public Health does not copyedit accepted manuscripts, so the language in submitted articles must be clear, correct, and unambiguous. Any typographical or grammatical errors should be corrected at revision, so please note any specific errors here.

Reviewer #1: Yes

**5. Review Comments to the Author**

Please use the space provided to explain your answers to the questions above. You may also include additional comments for the author, including concerns about dual publication, research ethics, or publication ethics. (Please upload your review as an attachment if it exceeds 20,000 characters)

Reviewer #1: This paper aimed to determine the prevalence and factors associated with overweight and obesity among women of reproductive age (WRA) in Cambodia. The authors used the Cambodia Demographic and Health Survey (CHDS) as the data source and, therefore were able to provide nationally representative data. Considering the rise of overweight/obesity among WRA in Cambodia (i.e. 18% in 2014 to 39% in 2021-2022), this study raised an important emerging issue in the country.

Thank.

Main comments:

- The first main concern is on Table 3. This analysis approach is referred as “Table 2 Fallacy”, in which all estimates were interpreted in the same way as total-effect estimates. Meanwhile, the interpretation of a confounder effect estimate may be different than for the exposure effect estimate (e.g. total effect for main exposure vs. direct effect for covariates). This is the key article explaining about this issue: https://www.ncbi.nlm.nih.gov/pmc/articles/PMC3626058/ , and here is a concrete example that quantitatively illustrate the potential of misinterpretation: https://pubmed.ncbi.nlm.nih.gov/29782045/. A Directed Acyclic Graph (DAG) that presents the conceptual framework of the study would help to carefully clarify this problem e.g. Have all potential confounders been considered? Are any of the factors on the causal pathway between one factor and the outcome? The current approach used in this paper should be acknowledged as substantial limitations to draw causal conclusions.

- Considering that the factors included in the analysis have been known associated with overweight/obesity, what does this paper add to the existing knowledge

1. The interpretation of a confounder effect estimate may be different from that of the exposure effect estimate using multiple logistic regression first following a statistically significant level at < 0.10 previously published by the authors: <https://pubmed.ncbi.nlm.nih.gov/37000710>. And potential confounder variables (for example, women’s age, wealth index, alcohol consumption, residence, and geographic region).
2. Multi-collinearity between right-hand-sided variables was checked using a continuous version of the outcome variable and evaluating VIF scores after fitting an Ordinary Least Squares regression model.
3. Effect modification of statistically significant associations by interaction models was estimated for each of the statistically significant factors from the adjusted analysis presented in Table 3.

Introduction:

- Authors could add explanations on trends that occurred in Cambodia, e.g. lifestyle changes, that may lead to the increase of overweight/obesity prevalence among WRA. Hence, this will highlight the importance of the study.

Thank. We have provided trends of behavioral risk increased risk of overweight and obesity in Cambodia including smokers of tobacco, and alcohol drinkers’ data from Cambodia STEP Survey 2010-2016.

- p.3 line 62: Why is the prevalence of overweight/obesity among WRA based on CDHS mentioned in the introduction (i.e. 39%) different from the study result (i.e. 22.56% overweight + 5.61% obesity = 28.17)?

The original CDHS 2021–22 has two separately calculated women's BMI. For example, for women aged **15–19** years old, BMI-for-age is measured in z-score standard deviations (SD) (WHO 2007), and for women aged **20–49** years old, BMI is calculated by dividing weight in kilograms by height in meters squared (kg/m^2^). However, in this study women's BMI is defined by dividing a person’s weight in kilograms by the square of their height in meters (kg/m^2^) for all women aged **15–49** years old following WHO standards, with restricted women samples.

- p.3 line 65-74: Are these predictors of overweight/obesity among WRA based on global data/other countries’ data?

There were predictors of overweight/obesity among WRA evidence from Demographic and Health Survey (DHS) data such as Cambodia, Bangladesh, Nepal, India, and Ethiopia.

Material and Methods:

Clear explanation on methods part. Below are minor feedback for this section:

- p.4 line 8-9: This “From September 15, 2021, to February 15, 2022” seems an incomplete sentence.

Thank. We have revised it accordingly.

- p.5 line 36: “Normal weight“ should be used instead of “average weight”.

Thank. We have revised it accordingly.

Results:

- The number of subjects included (n) can be restated at the beginning of the section.

- p.7 line 98: Missing percentage symbol on “18.60”

Thank. We have revised it accordingly.

- p.7 line 99: Should be 22.56% instead of 22,56%

Thank. We have revised it accordingly.

- Table 1: n of obese women had decimal i.e. 528.2 – can the author confirm this? Please check the consistency of using “()” instead of “[]” on the 95%CI in the BMI status rows.

Thank. We have revised it accordingly.

- p.9 line 27-43 and Table 2: This paragraph was written as if the authors did post-hoc analyses, which were not the case. For instance, it could not be concluded that women who used non-hormonal contraceptive methods had a significantly higher proportion of overweight/obesity than women using other methods (line 38); This is a type of conclusion that can be drawn from a posthoc analysis or if any regrouping were done. I wonder if that is the case since the p-value for contraceptive use as the predictor on page 10 line 238 (i.e. <0.019) is different from the p-value provided in Table 2 (i.e. 0.001).

Thank. We have corrected it as highlighted in the text. Also, we confirmed that the Table 2 result is performed by the bivariate analysis using the Chi-Square test.

- Please add an explanation for the use of bold text in certain numbers within Table 2

Thank. We have already explained the on-p.9 lines 26-43. For the proposed use of bold text in certain numbers within Table 2 just want to note or highlight the key indicators of crude associated with a high proportion of overweight/obesity in WRA.

Discussion:

- It needs more explanation on the findings in overweight/obesity prevalence e.g. how is this finding compared to previous studies/years and/or other countries? Why there were substantial differences in the prevalence across provinces (e.g. 34.1% in Kampong Cham vs. 9.4% in Ratanak Kiri)?

Thank. We have written more explanations of the variation and increase in overweight and obesity in Cambodia as the reviewer suggested.

- Based on the findings of the current analysis, it needs an explanation on how marital status emerged as a significant predictor of overweight/obesity.

Thank. We have written more explanations of this significant predictor as the reviewer suggested.

Conclusion:

Please clarify regarding the statement “design intervention programs that target these sociodemographic factors”. For instance, given that higher education levels and income are associated with overweight/obesity in this study – should women reduce their socioeconomic status? Similarly, since older age is linked to higher odds, should interventions aiming to reducing women’s age?

The policymakers should formulate and implement various awareness-raising initiatives regarding the adverse impact of overweight and obesity to higher risk groups such as older, married, wealthier, high parity and drinker alcohol women who are more prone to be overweight and obese and to raise awareness of the importance of consuming healthy food and the benefits of regular physical activity, especially recommended for among older women monitor their weight, blood pressure, and blood sugar with healthcare profession.

6. PLOS authors have the option to publish the peer review history of their article (what does this mean?). If published, this will include your full peer review and any attached files.

Do you want your identity to be public for this peer review? If you choose “no”, your identity will remain anonymous but your review may still be made public.

For information about this choice, including consent withdrawal, please see our Privacy Policy.

Reviewer #1: No
