# Supplementary material for: Factors associated with overweight and obesity among women of reproductive age in Cambodia: Analysis of Cambodia Demographic and Health Survey 2021–22
Source: PLOS Glob Public Health. 2024 Jan 31;4(1):e0002537. doi: 10.1371/journal.pgph.0002537 (PMC10830042; doi:10.1371/journal.pgph.0002537)
Supplement: Attachment — Submitted filename: Response to Reviewers.docx [file pgph.0002537.s004.docx]

**Authors’ point-by-point responses to the editor and reviewers**

PGPH-D-23-01290R1

Factors Associated with Overweight and Obesity among Women of Reproductive Age in Cambodia: Analysis of Cambodia Demographic and Health Survey

PLOS Global Public Health

Dear Dr. Um,

Thank you for submitting your manuscript to PLOS Global Public Health. After careful consideration, we feel that it has merit but does not fully meet PLOS Global Public Health’s publication criteria as it currently stands. Therefore, we invite you to submit a revised version of the manuscript that addresses the points raised during the review process.

=============================

Reviewer 2 corresponded with me to clarify that the manuscript isn’t “wrong” just there are aspects that are debatable or not comprehensive enough. Can you revise in response to their comments, and focus on more cautious interpretation of odds ratios, to make sure you are not claiming to prove a causal link with observational data?

==============================

Please submit your revised manuscript by Dec 30 2023 11:59PM. If you will need more time than this to complete your revisions, please reply to this message or contact the journal office at globalpubhealth@plos.org. When you're ready to submit your revision, log on to https://www.editorialmanager.com/pgph/ and select the 'Submissions Needing Revision' folder to locate your manuscript file.

Please include the following items when submitting your revised manuscript:

A rebuttal letter that responds to each point raised by the editor and reviewer(s). You should upload this letter as a separate file labeled 'Response to Reviewers'.

A marked-up copy of your manuscript that highlights changes made to the original version. You should upload this as a separate file labeled 'Revised Manuscript with Track Changes'.

An unmarked version of your revised paper without tracked changes. You should upload this as a separate file labeled 'Manuscript'.

Guidelines for resubmitting your figure files are available below the reviewer comments at the end of this letter.

We look forward to receiving your revised manuscript.

Kind regards,

Abraham D. Flaxman, Ph.D.

Academic Editor

PLOS Global Public Health

**Journal Requirements:**

1. Please review your reference list to ensure that it is complete and correct. If you have cited papers that have been retracted, please include the rationale for doing so in the manuscript text, or remove these references and replace them with relevant current references. Any changes to the reference list should be mentioned in the rebuttal letter that accompanies your revised manuscript. If you need to cite a retracted article, indicate the article’s retracted status in the References list and also include a citation and full reference for the retraction notice.

[Note: HTML markup is below. Please do not edit.]

**Reviewers' comments:**

**Reviewer's Responses to Questions**

Comments to the Author

1. If the authors have adequately addressed your comments raised in a previous round of review and you feel that this manuscript is now acceptable for publication, you may indicate that here to bypass the “Comments to the Author” section, enter your conflict of interest statement in the “Confidential to Editor” section, and submit your "Accept" recommendation.

Reviewer #2: (No Response)

2. Does this manuscript meet PLOS Global Public Health’s publication criteria? Is the manuscript technically sound, and do the data support the conclusions? The manuscript must describe methodologically and ethically rigorous research with conclusions that are appropriately drawn based on the data presented.

Reviewer #2: Partly

3. Has the statistical analysis been performed appropriately and rigorously?

Reviewer #2: No

4. Have the authors made all data underlying the findings in their manuscript fully available (please refer to the Data Availability Statement at the start of the manuscript PDF file)?

The PLOS Data policy requires authors to make all data underlying the findings described in their manuscript fully available without restriction, with rare exception. The data should be provided as part of the manuscript or its supporting information, or deposited to a public repository. For example, in addition to summary statistics, the data points behind means, medians and variance measures should be available. If there are restrictions on publicly sharing data—e.g. participant privacy or use of data from a third party—those must be specified.

Reviewer #2: Yes

5. Is the manuscript presented in an intelligible fashion and written in standard English?

PLOS Global Public Health does not copyedit accepted manuscripts, so the language in submitted articles must be clear, correct, and unambiguous. Any typographical or grammatical errors should be corrected at revision, so please note any specific errors here.

Reviewer #2: Yes

**6. Review Comments to the Author**

Please use the space provided to explain your answers to the questions above. You may also include additional comments for the author, including concerns about dual publication, research ethics, or publication ethics. (Please upload your review as an attachment if it exceeds 20,000 characters)

Reviewer #2: This manuscript investigated the factors associated with overweight and obesity in Cambodia using the latest Demographic and Health Survey (DHS) data. It focused on women of reproductive age and examined sociodemographic variables and selected behavioral risk factors such as use of contraceptives, smoking, and alcohol use. While the study provided some insight into the obesity epidemic in Cambodia, several aspects could be improved:

1. Factors associated with overweight and obesity: The authors acknowledged the omission of diet and physical activity from the study's considered factors, which are crucial direct influences on overweight and obesity outcomes. Although understanding the contribution sociodemographic factors to overweight and obesity is useful, it only offers a distal perspective, leaving a gap in understanding the most immediate factors.

Response: We thank the reviewer for this comment. For clarity, we are not omitting these variables. However, CDHS 2021-22 did not collect data on diet and physical activity. It fails to meet our study's limitations.

2. Goodness of fit: The manuscript does not clarify the extent to which the selected variables in the multiple logistic regression account for variability in the data. Questions remain regarding the fit of the regression model and the proportion of unexplained variance.

Response: We thank the reviewer for their original and additional observations regarding this issue. We agree with the reviewer’s observation in their additional feedback that is listed below regarding this issue of concern. We have revised to highlight that our primary inclusion criterion is theoretical relevance. The additional criteria of < 0.10 is used as we favor a more conservative level for the inclusion criterion.

3. Interpretation of AOR: Setting aside concerns of multicollinearity and model fit, the interpretation of results in Table 3 is convoluted. The odds ratios of several factors shifted from significant to non-significant after adjustment, with urban versus rural residency as an example. This change was likely due to the inclusion of other relevant factors such as occupation and wealth. However, the change of significance did not imply occupation being more important than place of residence. It was unclear, what conclusion should be drawn from the results in Table 3 comparing between OR and AOR.

Response: We have revised the manuscript to explain that concerns with Multicollinearity between independent variables were checked before fitting the final logistic regression model, including women's age, number of children ever born, education, household wealth index, occupation, marital status, place of residence, and geographical regions. The result of the evaluation of variance inflation factor (VIF) scores after fitting an Ordinary Least Squares regression model with the mean = 1.44 indicated no collinearity concerns.

4. Comparisons with the 2014 study: The study could benefit from a more detailed comparison with the DHS 2014 study. The current manuscript essentially replicated the previous study with new DHS data and the conclusion were similar. It would be informative to delineate the differences, changes over the decade, and any new insights provided by the latest research.

Response: We revised the manuscript conclusion toward a more direct comparison of comparable time periods.

Other comments:

- Page 4, line 120: The term "height and length" should be revised to "height" when referring to adults.

Thank. We have revised it accordingly.

- The general flow and coherence between sentences and paragraphs could be enhanced with editorial support.

Response: We have asked professional editing services to revise and make corrections grammatically and accordingly, as suggested.

7. PLOS authors have the option to publish the peer review history of their article (what does this mean?). If published, this will include your full peer review and any attached files.

Do you want your identity to be public for this peer review? If you choose “no”, your identity will remain anonymous but your review may still be made public.

For information about this choice, including consent withdrawal, please see our Privacy Policy.

Reviewer #2: No
